# Orbital Blowout Fracture with Complete Dislocation of the Globe into the Maxillary Sinus

**DOI:** 10.7759/cureus.1728

**Published:** 2017-09-29

**Authors:** Joy MH Wang, Fabian N Fries, Philipp Hendrix, Titus Brinker, Marios Loukas, R. Shane Tubbs

**Affiliations:** 1 Department of Anatomical Sciences, St. George's University School of Medicine, Grenada, West Indies; 2 Ophthalmology, Saarland University Medical Center, Germany; 3 Neurosurgery, Saarland University Medical Center, Germany; 4 Dermatology, University Hospital Essen; 5 Department of Anatomical Sciences, St. George's University School of Medicine, Grenada, West Indies; 6 Neurosurgery, Seattle Science Foundation

**Keywords:** low velocity trauma, isolated orbital floor fracture, orbital blow-out fracture, orbit, eye, oculoplastic surgery, adolescent facial trauma, globe dislocation

## Abstract

This rare case report describes the diagnosis and treatment of an isolated left-sided orbital floor fracture with a complete dislocation of the globe into the maxillary sinus and briefly discusses the indications of surgery and recovery for orbital floor fractures in general. Complete herniation of the globe through an orbital blow-out fracture is uncommon. However, the current case illustrates that such an occurrence should be in the differential diagnosis and should be considered, especially following high speed/impact injuries involving a foreign object. In these rare cases, surgical intervention is required.

## Introduction

Fractures of the orbit can occur in isolation or in association with more complex fracture patterns and globe dislocations. Operative intervention of adult orbital floor fractures is generally reserved for fractures producing an alteration in vision and/or clinically detectable aesthetic deformities. Generally accepted guidelines include a fracture involving more than 50% of the orbital floor and/or fractures larger than 2 cm^2^ [1, 2]. These recommendations are based, in part, on multiple studies that demonstrate the correlation between fracture size and the development of clinically detectable enophthalmos and/or visual disturbances. Certain presentations such as acute diplopia and/or muscle entrapment dictate operative intervention [3-6]. However, less is known about the long-term visual and aesthetic outcomes of orbital floor fractures. Herein, we report an unusual case of orbital blow-out fracture following acute trauma in an adolescent boy.

## Case presentation

**Figure 1:**
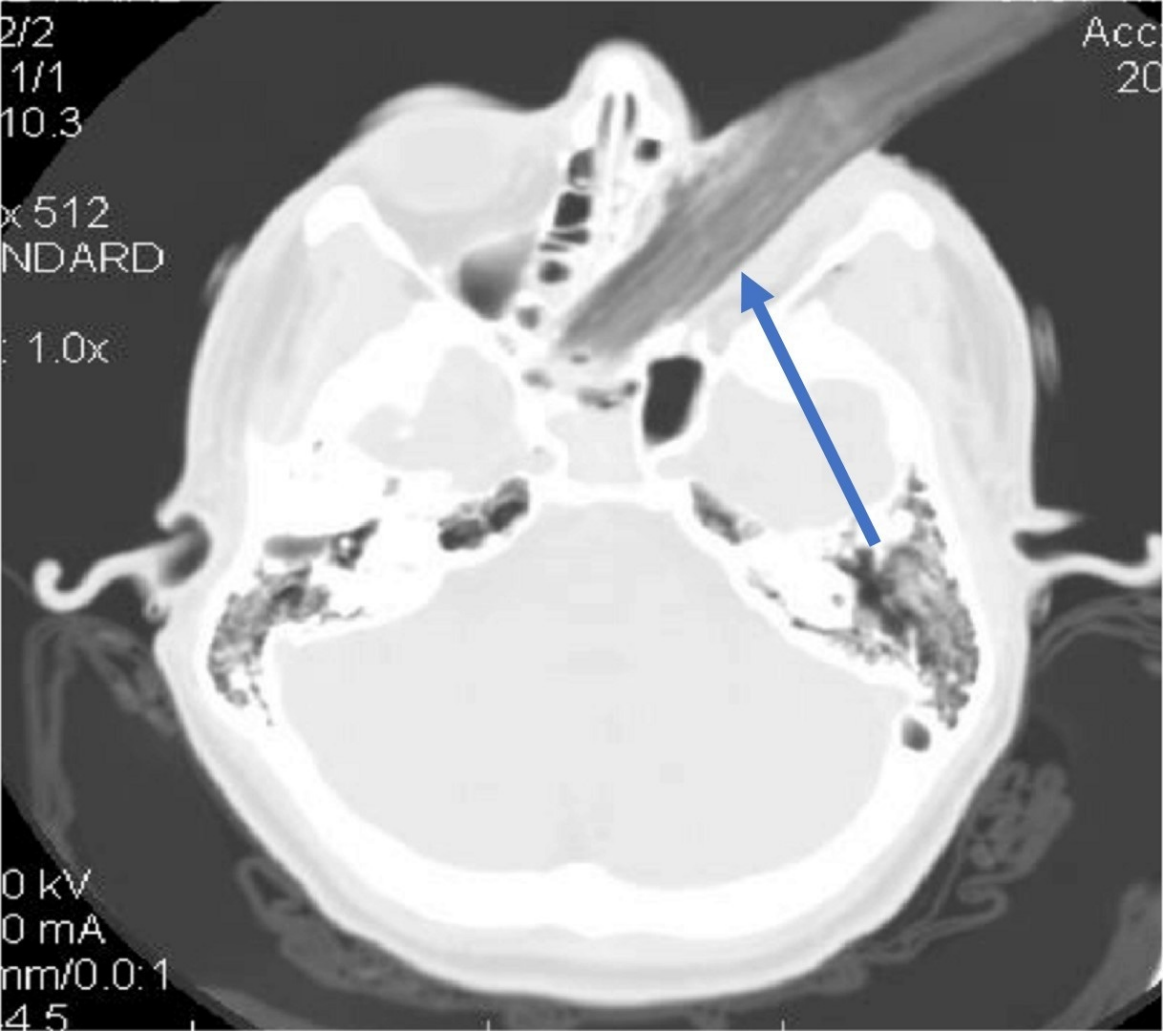
Computed tomography (CT) of head: axial view through the orbit. Blue arrow: tree branch in the left orbit extending into the nasal cavity, ethmoidal air cells, and right sphenoid sinus via the medial orbital wall.

**Figure 2:**
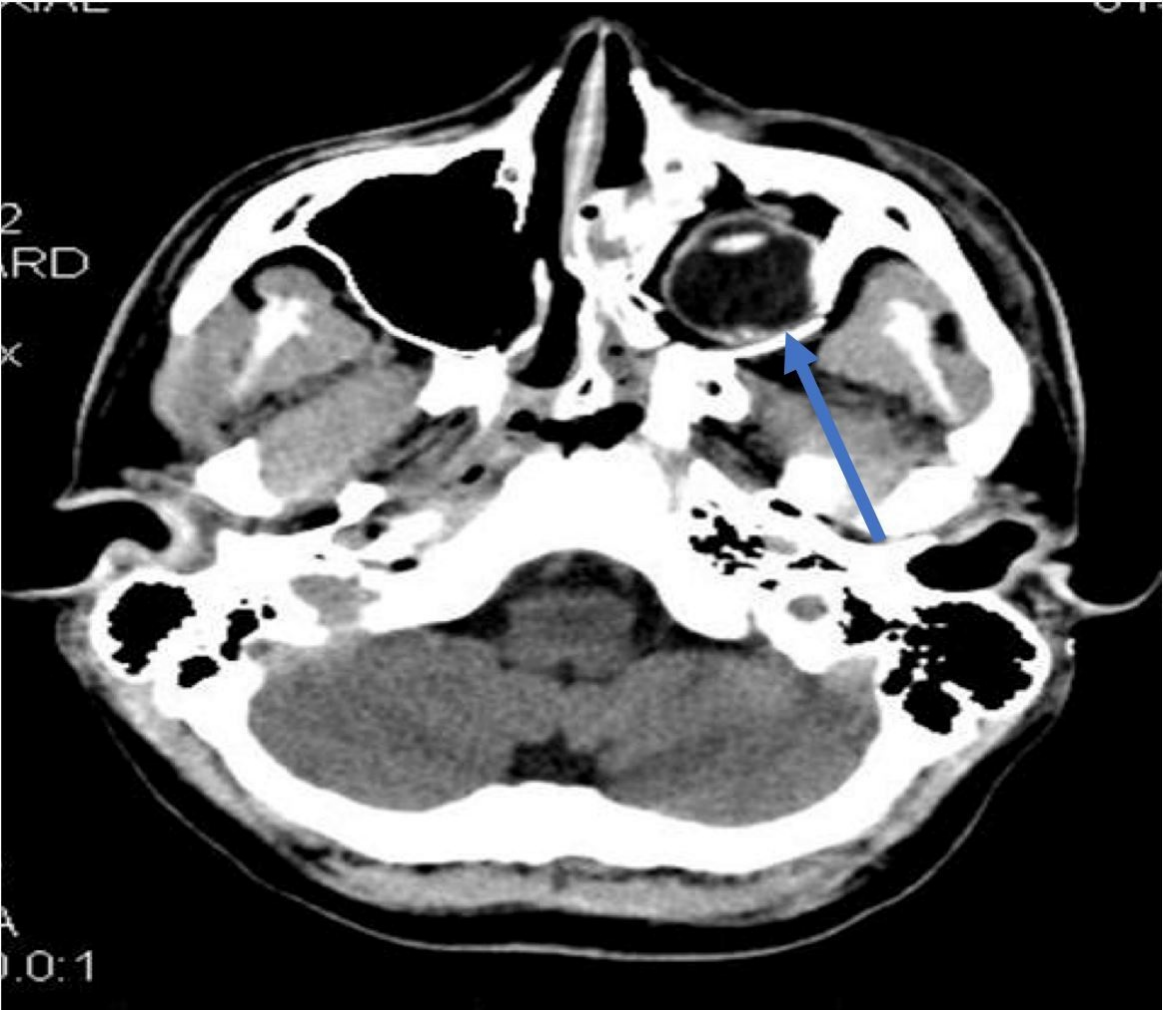
Computed tomography (CT) of head: axial view through the maxillary sinus. Blue arrow: the globe with intact lens sitting within the maxillary sinus.

A 15-year-old boy presented to the emergency department after a penetrating injury to his left eye caused by driving an all-terrain vehicle (ATV) without a helmet. At presentation, a large tree branch was lodged into his left orbit (Figure 1). He was in severe pain, but conscious and able to move all extremities. He had normal vision in the right eye but denied vision in the left. Head computed tomography (CT) displayed a left orbital blow-out fracture. Axial cuts through the ipsilateral maxillary sinus, showed a foreign object – an ectopically positioned left globe (Figure 2). The tree branch violated the medial left orbital wall and entered the nasal cavity. Contralateral vision and extraocular muscle functions were normal, with no other injuries found. In surgery, the tree branch was removed and the penetrating pathway explored using a transconjunctival approach. The fracture was identified, the optic nerve was completely severed, but the globe and lens were relatively intact. Titanium mesh was used to repair the orbital floor and medial walls. Postoperative course was uncomplicated. At one-year follow-up, the patient was doing well, without new neurological deficits nor issues with his ocular prosthesis.

## Discussion

Ambiguity prevails in the medical literature regarding the necessity and ideal timing of surgical intervention to treat orbital floor fractures. Indications for surgery include the “white-out” orbital floor fracture, that despite little evidence of trauma, leads to restriction of upward gaze due to entrapment of the inferior rectus muscle; this also often triggers the oculocardiac reflex, resulting in nausea, vomiting, bradycardia, and hypotension. Trauma-induced orbital fractures often present with subconjunctival hemorrhage, enophthalmos, swollen lid, ecchymosis and preretinal hemorrhage due to the temporary increased pressure. An Nd:YAG laser hyaloidotomy represents a minimally invasive treatment for the latter complication providing fast vision acuity recovery [7]. With these fractures, dislocation of orbital contents can also occur in various degrees; albeit rare, complete dislocation of the globe into the maxillary sinus can occur. These cases necessitate surgical intervention to both explore the extent of injury and repair the damage. Recovery is variable, with the extent and mechanism of injury playing significant roles. Typically, when the eye is struck by a blunt blow, as with a fist or rock, the globe remains intact, while the orbital wall fractures [8]. In the cases described by Kohlhof and Berkowitz, both patients suffered similar initial presentations, fractures, and recoveries [8, 9]; vision in the affected eye was salvaged, as the integrity of the globe, continuity of the optic nerve, and intactness of the surrounding structures were preserved. These patients recovered with only mild anisocoria and mildly diminished globe motility in the affected eye. In contrast, as with Ginat’s and our patient, a penetrating injury causing clear evidence of damage to the integrity of the globe and/or continuity of the optic nerve, rendered the vision in the affected eye unsalvageable [10]. Thus, it appears that penetrating injuries to the eye resulting in globe dislocation have a higher incidence of permanent vision loss; however, in all these cases of orbital floor fractures with complete globe dislocation, the indication for surgical intervention is indisputable.

## Conclusions

Complete herniation of the globe through an orbital blow-out fracture is uncommon. However, the current case illustrates that such an occurrence should be in the differential diagnoses, especially following high speed/impact injuries involving a foreign object. These rare cases require surgical intervention. Patients can recover well with good functional result, with recovery of vision largely determined by the extent of damage to the globe integrity and optic nerve continuity.
